# Maternal supplementation of diabetic mice with thymoquinone protects their offspring from abnormal obesity and diabetes by modulating their lipid profile and free radical production and restoring lymphocyte proliferation via PI3K/AKT signaling

**DOI:** 10.1186/1476-511X-12-37

**Published:** 2013-03-18

**Authors:** Gamal Badr, Mohamed H Mahmoud, Karim Farhat, Hanan Waly, Osman Zin Al-Abdin, Danny M Rabah

**Affiliations:** 1 Present address: Princess Al-Johara Al-Ibrahim Center for Cancer Research, Prostate Cancer Research Chair, College of Medicine, King Saud University, Riyadh, Saudi Arabia; 2 Zoology Department, Faculty of Science, Assiut University, 71516 Assiut, Egypt; 3 Human Nutrition Department, National Research Centre, Dokki, Cairo, Egypt; 4 Deanship of Scientific Research, King Saud University, Riyadh, Saudi Arabia; 5 Department of Urology/Surgery, College of Medicine, King Saud University, Riyadh, Saudi Arabia

**Keywords:** Maternal diabetes, Offspring, Obesity, T cell immune response, PI3K/AKT signaling

## Abstract

**Background:**

Epidemiological studies have shown that the offspring of mothers who experience diabetes mellitus during pregnancy are seven times more likely to develop health complications than the offspring of mothers who do not suffer from diabetes during pregnancy. The present study was designed to investigate whether supplementation of streptozotocin (STZ)-induced diabetic pregnant mice with thymoquinone (TQ) during pregnancy and lactation improves the risk of developing diabetic complications acquired by their offspring.

**Methods:**

Three groups of pregnant female mice were used: non-diabetic control dams (CD), diabetic dams (DD), and diabetic dams supplemented with TQ (DD + TQ) during pregnancy and lactation (n = 10 female mice in each group).

**Results:**

Our data demonstrated a marked decrease in the number of neonates born to DD, and these neonates showed a marked increase in their mean body weight (macrosomic pups) compared to those born to CD and DD + TQ. The induction of diabetes during pregnancy and lactation resulted in macrosomic pups with several postpartum complications, such as a marked increase in their levels of blood glucose, free radicals, plasma pro-inflammatory cytokines (IL-1β, IL-6, and TNF-α), and lipids, and a tendency toward abnormal obesity compared to the offspring of CD. By contrast, macrosomic offspring born to DD exhibited a marked reduction in plasma cytokine levels (IL-2, -4 and -7), an obvious reduction in the number of circulating lymphocytes, decreased proliferation of superantigen (SEB)-stimulated lymphocytes and aberrant AKT phosphorylation. Interestingly, the supplementation of DD with TQ during pregnancy and lactation had an obvious and significant effect on the number and mean body weight of neonates. Furthermore, TQ significantly restored the levels of blood glucose, insulin, free radicals, plasma cytokines, and lipids as well as lymphocyte proliferation in the offspring.

**Conclusions:**

Our data suggest that the nutritional supplementation of DD with the natural antioxidant TQ during pregnancy and lactation protects their offspring from developing diabetic complications and preserves an efficient lymphocyte immune response later in life.

## Background

Gestational diabetes mellitus (GDM) is defined as diabetes with initial onset during pregnancy [1]. Maternal hyperglycemia gives rise to complications for both the mother and child. These complications include macrosomia, problems with delivery that are usually associated with fetal overgrowth, and increased rates of caesarean section [2]. Furthermore, obesity appears to be another long-term complication in offspring born to mothers with GDM [3]. In particular, a recent rat study revealed that the offspring of diabetic dams exhibited abnormal changes in their lipid profile as well as metabolic disorders that can affect the growth and metabolism of their descendants and subsequent generations [4]. In addition, the offspring of women with diabetes during pregnancy are at increased risk of developing hypertension and other cardiovascular diseases [5]. Insulin resistance and insulin secretion, the metabolic predictors of diabetes development, have been investigated in the offspring of diabetic mothers in humans and animal models [6]. Because insulin from the mother does not cross the placenta, the pancreatic insulin output of the fetus is solely determined by the glucose levels in the maternal blood [7]. Evidence suggests that prenatal exposure to a diabetic intrauterine environment is associated with increased risk for impaired glucose tolerance [8] and type 2 diabetes among offspring [9]. The involvement of oxidative stress is one of the earliest abnormalities observed in diabetic subjects [10]. Moreover, fetuses from mothers with gestational diabetes are at increased risk of developing oxidative stress, which subsequently induces the production of highly reactive oxygen radicals, which are toxic to cells, particularly to the plasma membrane, where these radicals interact with the lipid bilayer [11]. In humans it has been shown that when compared with non-diabetic women, women with GDM exhibit a decreased concentration of adiponectin (an anti-inflammatory agent) and an increased concentration of TNF-α and IL-6 (pro-inflammatory cytokines) [12]. By contrast, serum IL-4 levels do not differ between gestational diabetic mothers and control mothers or macrosomic babies and control babies [13]. High levels of pro-inflammatory cytokines have also been implicated as major mediators of demyelination in the central nervous system, resulting in a variety of inflammatory and neoplastic diseases, and many other pathological complications [14]. By contrast, a decrease in the plasma levels of IL-2 and IL-7 (T cell growth factors that promote T cell survival) occurs in several diseases and reflects defective T cell function [15]. In addition, recent studies have indicated that GDM mothers and their newborns have lymphocyte subset impairments, which are more important in patients who are positive for autoantibodies and/or treated with insulin [16]. We previously demonstrated in an animal model that neonates born to diabetic dams exhibit a marked reduction in the proliferative capacity of superantigen (SEB)-stimulated T-lymphocytes and an obvious reduction in the number of circulating and thymus homing T cells [17].

The beneficial effects of antioxidant supplementation are attributed to the ability of these molecules to scavenge free radicals, control nitric oxide synthesis or release, inhibit reactive oxygen species generation, and upregulate the activity of the antioxidant enzymes that metabolize these molecules [18]. Plant-based antioxidants, such as black seeds, have recently gained popularity due to their role as dietary supplements that have minimal side effects. Thymoquinone (TQ) is the major component of the essential oil of black seeds and the biologically active ingredient [19]. TQ has been reported to exhibit many pharmacological effects, including immunomodulatory [20], anticancer [21], anti‐diabetic [22], antioxidant, and anti‐inflammatory [23] activities. TQ regulates the plasma concentrations of cholesterol, triglycerides, and glucose [19]. Despite studies that have demonstrated strong evidence for the antioxidative effects of different natural products, there remains insufficient information concerning the effects of TQ on developing diabetic complications and, in particular, on the T cell immune response in GDM. Therefore, the present study was designed to investigate whether supplementation with TQ during pregnancy and lactation periods to streptozotocin (STZ)-induced diabetic pregnant mice improves the developed diabetic complications in their offspring.

## Materials and methods

### Chemicals

STZ was obtained from Sigma Chemicals Co. (St. Louis, MO, USA). STZ was dissolved in cold 0.01 M citrate buffer (pH 4.5) and was always prepared freshly for immediate use within 5 min. TQ was obtained from Sigma Chemical Company (St Louis, MO, USA).

### Experimental animal model

A total of 60 laboratory-bred Swiss albino mice of either sex, 6-8 weeks of age and weighing 25-30 g were obtained from the Faculty of Medicine of Assiut University (Assiut, Egypt). All animal procedures were in accordance with the standards of the National Institutes of Health (NIH). The study protocol was approved by the Animal Ethical Committee of Assiut University. The animals were allowed to acclimatize for 2 weeks before the experiment. The animals were housed in metal cages inside a well-ventilated room. Each cage contained no more than 2 mice (2 males or 2 females). The mice were maintained under standard laboratory conditions: a temperature of 25°C, a relative humidity of 60-70%, and a 12-hour light/dark cycle. The mice were fed a standard commercial pellet diet and water.

All female mice were fasted for 20 h before diabetes was induced with STZ. Mice (n = 30) were given 5 consecutive daily i.p. injections of STZ (50 mg/kg body weight) in 0.1 M citrate buffer (pH 4.5) before mating began. The animals were assigned to one of 2 experimental groups (15 mice per group): one group received only STZ, while the other received STZ and was orally supplemented with TQ (20 mg/kg body weight/day) during gestation and lactation. A control naive group (n = 15) was injected with vehicle alone (0.1 M citrate buffer [pH 4.5]) and orally supplemented with distilled water (150 μl/mouse/day) during gestation and lactation. After mating, the first day of gestation was determined by the presence of spermatozoids in the vaginal smears. Pregnant mice were housed individually in metal cages under the above-mentioned conditions. To assess hyperglycemia during gestation, the tip of the tail was cut off, and the tail was squeezed gently to collect blood. Blood glucose levels were measured once per week after overnight fasting, beginning with the day of the STZ injection until 2 weeks postpartum using an AccuTrend sensor (Roche Biochemicals, Mannheim, Germany). The data were used to calculate the mean blood glucose levels for each group, and mice were considered diabetic if glycemia was higher than 220 mg/dl. The neonates were h.

### Blood analysis

At the end of the experiment, the 8-week-old offspring in each group were anesthetized with pentobarbital (60 mg/kg body weight), the abdominal cavity was opened, and whole blood was drawn from the abdominal aorta. Plasma was obtained by low-speed centrifugation (1,000 × *g* for 20 min) and immediately stored at -80°C for subsequent cytokine profile analysis. PBMCs were also isolated using the Ficoll gradient method. Insulin levels were analyzed by Luminex (Biotrend, Düsseldorf, Germany) according to the manufacturer’s instructions.

### Measurement of free radical levels

The levels of reactive oxygen species (ROS) were determined using 2,7-dichlorodihydrofluorescein diacetate (H2DCF-DA) (Beyotime Institute of Biotechnology, Haimen, China). Hydroperoxide levels were evaluated using a free radical analytical system (FRAS 2, Iram, Parma, Italy). This test is a colorimetric test that takes advantage of the ability of hydroperoxide to generate free radicals after reaction with transition metals.

### Lipid profile analysis

Lipid profiles were determined using BioMerieux kits and a standard assay method. Cholesterol levels were evaluated using the cholesterol esterase method. Triglycerides were measured using the lipase method. HDL, LDL, and chylomicrons were precipitated with phosphotungstic acid. The amount of cholesterol bound to HDL was determined using the cholesterol oxidase method and the phosphotungstate-magnesium salt method using a Cholesterol E-Test Kit (Wako, Osaka, Japan) as previously described [24].

### Determination of plasma cytokine levels

Cytokine levels were determined in samples that were stored at -80 C. Plasma cytokine (IL-1β, IL-2, IL-4, IL-6, IL-7, and TNF-α) levels were determined by ELISA using a Bio-Plex mouse cytokine assay kit (Bio-Rad, Hercules, CA, USA) according to the manufacturer’s instructions.

### CFSE proliferation assay

Peripheral blood mononuclear cells (PBMCs) were isolated from blood using the Ficoll gradient method. The PBMCs were then re-suspended at 20 × 10^6^ cells/ml in 1X phosphate buffered saline (PBS) and stained with 0.63 μM carboxyfluorescein diacetate succinimidyl ester (CFSE) (Molecular Probes, Eugene, OR, USA) for 8 min at room temperature. The reaction was stopped with FBS, and the cells were washed 3 times in PBS and resuspended at 2 × 10^6^ cells/ml in prewarmed R-10 medium. The CFSE-labeled cells were stimulated for 6 days with or without Staphylococcal enterotoxin B (SEB) (final concentration of 5 ng/ml) at 37°C and 5% CO_2_. On day 6, lymphocyte proliferation was analyzed by flow cytometry.

### Western blot analysis

Isolated PBMCs were pretreated with medium, wortmannin (WM; inhibitor of PI3K), and SH5 (inhibitor of AKT phosphorylation) for 1 h before stimulation with or without CXCL12 (250 ng/ml) for 5 min. Whole-cell lysates were prepared from the PBMCs in RIPA buffer (20 mM Tris-HCl [pH 7.5], 120 mM NaCl, 1.0% Triton X-100, 0.1% SDS, 1% sodium deoxycholate, 10% glycerol, 1 mM EDTA, and 1% protease inhibitor cocktail [Roche]). Following centrifugation at 16,000 × *g* for 15 min at 4°C, the protein concentration of each supernatant was determined using a protein assay kit (Bio-Rad, Hercules, CA). Equal amounts of each whole-cell protein lysate (50 μg) were mixed with reducing sample buffer (0.92 M Tris-HCl [pH 8.8], 1.5% SDS, 4% glycerol and 280 mM 2-mercaptoethanol) and separated by discontinuous SDS-PAGE. The proteins were then transferred onto nitrocellulose membranes using a Bio-Rad Trans-Blot electrophoretic transfer device. Next, the membranes were blocked for 1 h at room temperature with 1% BSA or 5% skim milk dissolved in TBS (20 mM Tris-HCl [pH 7.4] and 150 mM NaCl) supplemented with 0.1% Tween 20 and then incubated in the same blocking buffer with an anti-phospho-PKB/AKT (S473) or pan-AKT antibody (1:5,000; Cell Signaling). The blots were thoroughly rinsed and then incubated with an HRP-labeled species-matched secondary antibody for 1 h. Protein bands were detected by enhanced chemiluminescence (ECL, SuperSignal West Pico Chemiluminescent Substrate, Perbio, Bezons, France), and the ECL signals were recorded on Hyperfilm ECL. To quantify the protein band intensities, the films were scanned, saved as TIFF files, and analyzed using NIH ImageJ software.

### Statistical analysis

The data were tested for normality using the Anderson-Darling test and for homogeneity variances prior to further statistical analysis. The data were normally distributed and are expressed as the mean ± standard error of the mean (SEM). Significant differences among groups were analyzed by one- or two-way ANOVA followed by Bonferroni’s test for multiple comparisons using PRISM statistical software (GraphPad Software). The data were also reanalyzed by one- or two-way ANOVA followed by Tukey’s posttest using SPSS software (version 17). Differences were considered statistically significant at P < 0.05. ^*^P < 0.05 for diabetic vs. control; ^#^P < 0.05 for diabetic + TQ vs. diabetic; ^+^P < 0.05 for diabetic + TQ vs. control.

## Results

### Characteristics of gestational diabetes and its complications in offspring

The treatment of pregnant mice with STZ resulted in marked hyperglycemia that was still detectable during lactation. The success rate of pregnancy among the dams was clearly decreased by the induction of diabetes (Table 1). The induction of gestational diabetes (GD) before mating was associated with a decreased number of delivered neonates (64 pups born to DD vs. 121 pups born to CD) (Table 1). Treatment of DD with TQ during pregnancy and lactation had a clear effect on the total number of delivered neonates (96 pups vs. 64 in DD that were not treated with TQ). Furthermore, at 4 weeks of age, many of the pups born to DD that had been treated with TQ were still alive (85 pups vs. 48 pups born to DD that were not treated with TQ vs. 115 pups born to CD) (Table 1). Offspring born to DD typically presented higher body weights (macrosomic neonates) at birth compared to neonates born to DD treated with TQ and those born to CD. At 8 weeks of age, although the percentage of mortality was high among the pups born to DD (Table 2), these pups sustained a significantly higher body weight compared to pups born to DD treated with TQ and those born to CD. Moreover, at 8 weeks of age, the blood glucose and free radical levels (ROS and hydroperoxide) were significantly higher in the plasma of pups born to DD compared to pups born to DD treated with TQ and those born to CD. By contrast, offspring born to DD exhibited a significant reduction in circulating lymphocyte counts and an obvious decrease in insulin levels compared to offspring born to DD treated with TQ and those born to CD (Table 2).

**Table 1:** TQ supplementation improves materno-fetal parameters and neonatal outcome of pregnant diabetic dams

			**Control dams**	**Diabetic dams**	**Diabetic dams supplemented with TQ**
**Parameters**					
Total pregnancies			15	13	14
Successful pregnancies			14	9	12
Abortion (%)			6.66	31	14
Total neonates			121	64	96
Alive pups at four weeks			115	48	85
Dead pups			6	16	11
Mortality (%)			4.96	25	11.46
**Blood glucose (mg/dl)**	Before pregnancy	3d after vehicle or STZ injection	178 ± 14.5	265 ± 19.7^*^	259 ± 17.4^+^
	During pregnancy	1^st^ W	193 ± 17.1	283 ± 21^*^	247 ± 23^#+^
		2^nd^ W	203 ± 18.7	311 ± 23.5^*^	257 ± 16.9^#+^
		3^rd^ W	187 ± 12.4	342 ± 28^*^	266 ± 23.5^#+^
	After pregnancy	7d postpartum	179 ± 12.5	295 ± 28.4^*^	246 ± 19.3^#+^

The results are expressed as the mean ± SEM. ^*^P < 0.05 for diabetic vs. control; ^#^P < 0.05 for diabetic + TQ vs. diabetic; ^+^P < 0.05 for diabetic + TQ vs. control.

**Table 2:** Significant alterations in body weight, insulin level, lymphocyte counts, and levels of free radicals in offspring at 8 weeks of age

**Parameters**	**Offspring of control dams**	**Offspring of diabetic dams**	**Offspring of diabetic dams supplemented with TQ**
**At birth**			
Body weight (g)	1.24 ± 0.1	1.89 ± 0.1^*^	1.39 ± 0.11^#^
**At age of 8 weeks**			
body weight (g)	20 ± 1.9	28.9 ± 2.9^*^	23.3 ± 2.3
Blood glucose (mg/dl)	141 ± 12	218 ± 19.5^*^	178.5 ± 13.4^#+^
Insulin (ng/ml)	2.4 ± 0.1	1.52 ± 0.15^*^	2.1 ± 0.19^#^
WBCs count (10^3^/ul)	15 ± 0.9	11 ± 0.1^*^	13.5 ± 0.18^#^
Lymphocytes (%)	75 ± 5.8	58 ± 4.8^*^	71.6 ± 0.18^#^
Monocytes (%)	5 ± 0.18	4 ± 0.35	5 ± 0.6
Neutrophils (%)	20 ± 1.45	19 ± 1.3	21 ± 1.95
ROS (nmol/ml)	11 ± 1.9	25 ± 3.9^*^	17 ± 2.1^#+^
Hydroperoxide (mg/100 ml)	17 ± 2.7	34 ± 4.4^*^	24 ± 2.8^#+^

The results are expressed as the mean ± SEM. ^*^P < 0.05 for diabetic vs. control; ^**#**^P < 0.05 for diabetic + TQ vs. diabetic; ^**+**^P < 0.05 for diabetic + TQ vs. control.

### Offspring born to DD exhibits elevation in the lipid profiles with a tendency for abnormal obesity

**Figure 1:**
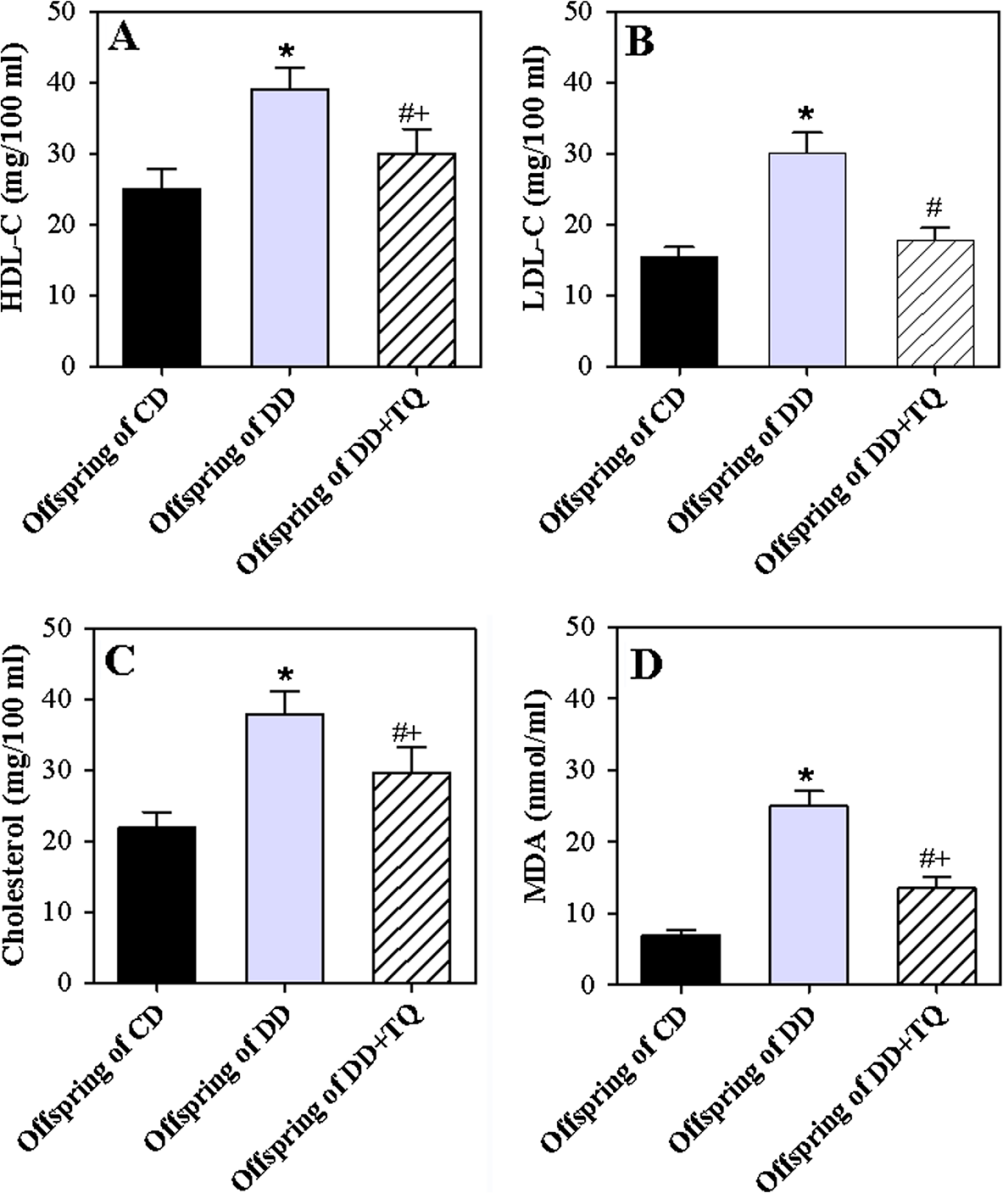
**TQ restores the lipid profile in the offspring of diabetic dams. **The levels of HDL-C (**A**), LDL-C (**B**), cholesterol (**C**), and MDA (**D**) were determined in offspring (8 weeks old) born to control dams (CD; black bars), diabetic dams (DD; gray bars), and diabetic dams supplemented with TQ (DD + TQ; hatched bars) as described in the Materials and Methods section. The accumulated data from 10 individual mice from each group are expressed as the mean level of each lipid ± SEM. ^*^P < 0.05 for diabetic vs. control; ^**#**^P < 0.05 for diabetic + TQ vs. diabetic; ^**+**^P < 0.05 for diabetic + TQ vs. control.

We previously observed that offspring born to DD typically presented higher body weights (macrosomic neonates) at birth and sustained a significantly higher body weight at 8 weeks of age compared to pups born to DD treated with TQ and those born to CD (Table 2). We therefore monitored the lipid profiles of the offspring of the 3 groups at 8 weeks of age. The levels of HDL-C (Figure 1A), LDL-C (Figure 1B), and cholesterol (Figure 1C) were significantly higher in the plasma of pups born to DD compared to pups born to DD treated with TQ and those born to CD. Moreover, MDA is a marker of oxidative lipid damage and a major oxidative product of peroxidized polyunsaturated fatty acids. The level of MDA was significantly increased in the plasma of pups born to DD compared to pups born to DD treated with TQ and those born to CD (Figure 1D). Supplementation of DD with TQ during pregnancy and lactation obviously restored the plasma lipid profiles of their offspring compared to those born to DD that were not treated with TQ.

### Supplementation of DD with TQ during GD restores the plasma cytokine profiles in their offspring

**Figure 2:**
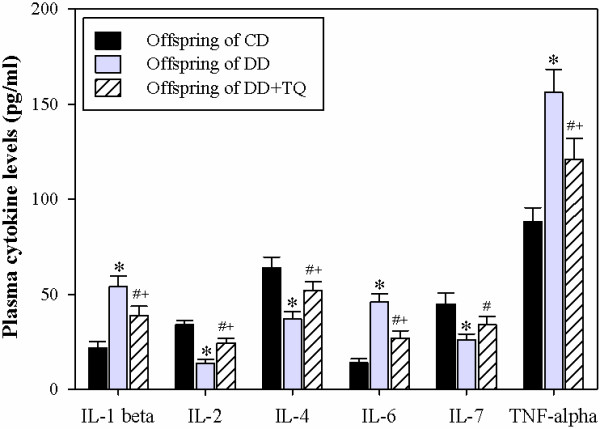
**TQ restores plasma cytokine levels in the offspring of diabetic dams. **The levels of plasma cytokines were measured in freshly isolated blood from offspring (8 weeks old) born to CD (black bars), DD (gray bars), and DD + TQ (hatched bars) as described in the Materials and Methods section. The accumulated data from 10 individual male mice from each group are expressed as the mean level of each cytokine (pg/ml) ± SEM. ^*^P < 0.05 for diabetic vs. control; ^**#**^P < 0.05 for diabetic + TQ vs. diabetic; ^**+**^P < 0.05 for diabetic + TQ vs. control.

Cytokines are secreted by specific cells of the immune system, carry signals locally between cells, and are critical for the development and function of both the innate and adaptive immune responses. Plasma cytokine levels of the pro-inflammatory cytokines IL-1β, IL-6 and TNF-α, the T cell growth factor IL-2, and the B-cell stimulatory factor-1 IL-4 were evaluated in the offspring at 8 weeks of age. The levels of the pro-inflammatory cytokines IL-1, IL-6, and TNF-α were significantly elevated in the offspring of DD compared to the offspring of CD (Figure 2). Nevertheless, treatment of DD with TQ during pregnancy and lactation significantly restored the levels of IL-1β, IL-6, and TNF-α in their offspring compared to those born to DD that were not treated with TQ. By contrast, the plasma levels of IL-2 (a T cell growth factor required for the survival and proliferation of T cells) and IL-4 (a cytokine secreted by activated T cells that induces the activation and differentiation of B cells) were significantly reduced in the offspring of DD compared to those of CD and DD treated with TQ.

### Treatment of DD with TQ during GD enhances SEB-mediated lymphocyte proliferation in their offspring

**Figure 3:**
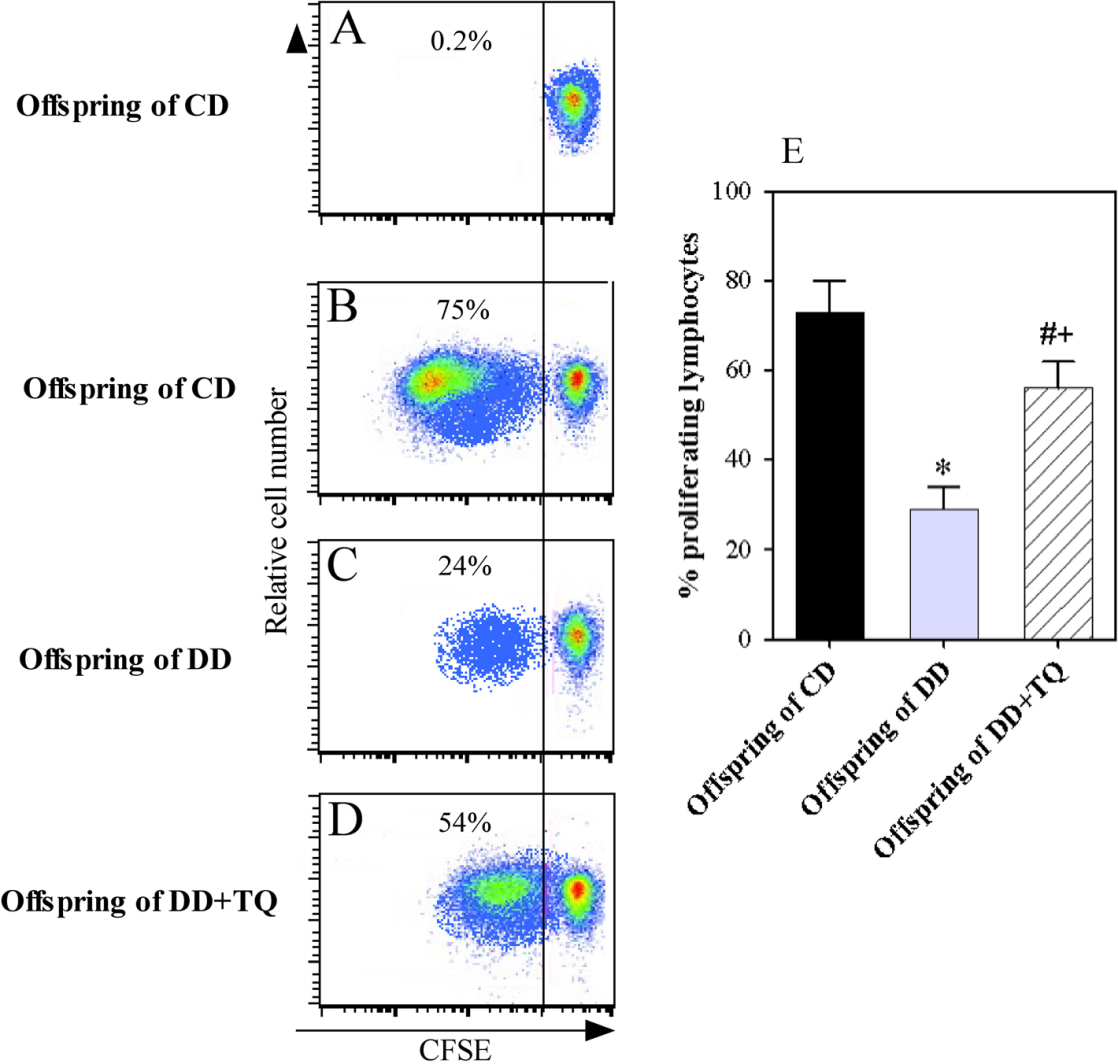
**TQ supplementation restores lymphocyte proliferation upon antigen stimulation.** PBMCs were isolated from the blood of offspring (8 weeks old) born to CD, DD, and DD + TQ, and their proliferative capacity was assessed in response to SEB following 6 days of stimulation using CFSE dilution assays and flow cytometry analysis. The plots were gated on unstimulated (**A**) or SEB-stimulated lymphocytes (**B**) of offspring born to CD, SEB-stimulated lymphocytes of offspring born to DD (**C**)**,** and SEB-stimulated lymphocytes of offspring born to DD (**D**) and were then gated on viable cells to exclude dead cells. The numbers on the left side represent the percentage of CFSE-lo (proliferating cells) within the cell population. One representative experiment is shown. (**E**) The data obtained for offspring (n = 10) born to CD (black bars), DD (gray bars), and DD + TQ (hatched bars) are expressed as the mean percentages of proliferating lymphocytes ± SEM. ^*^P < 0.05 for diabetic vs. control; ^**#**^P < 0.05 for diabetic + TQ vs. diabetic; ^**+**^P < 0.05 for diabetic + TQ vs. control.

Because decreased levels of IL-2 and IL-4 could induce a reduction in lymphocyte proliferation, we examined whether the lymphocytes of the offspring exhibited an exhausted status after superantigen stimulation using a CFSE dilution assay. PBMCs were isolated from offspring of CD, DD, and DD treated with TQ. The cells were then labeled with CFSE, stimulated with SEB, cultured for 6 days, and analyzed by flow cytometry. The plots were gated on lymphocytes according to the forward and side scatter and then on viable cells to exclude dead cells. The percentages of CFSE-lo (proliferating cells) and CFSE-high (non-proliferating cells) within the lymphocyte population are presented as dot plots (Figure 3). In one representative experiment, we determined that the percentage of lymphocyte proliferation was markedly increased from 0.2% in medium-treated cells to 75% in SEB-treated cells in the offspring of CD (Figures 3A &3B). The offspring of DD exhibited a marked reduction (24%) in the proliferative capacity of their lymphocytes (Figure 3C), and the percentage of proliferating lymphocytes was markedly restored (54%) in the offspring born to DD that were treated with TQ (Figure 3D). Accumulated data from 10 separate experiments with 10 offspring in each group revealed that the percentage of proliferating lymphocytes was significantly reduced from 73 ± 7% in the offspring of CD to 29 ± 5% in the offspring of DD (Figure 3E). Interestingly, the percentage of proliferating lymphocytes was significantly restored to 56 ± 6.4% in the offspring of DD that were treated with TQ.

### Treatment of DD with TQ during GD restores the impaired PI3K/AKT signaling pathway in the lymphocytes of their offspring

**Figure 4:**
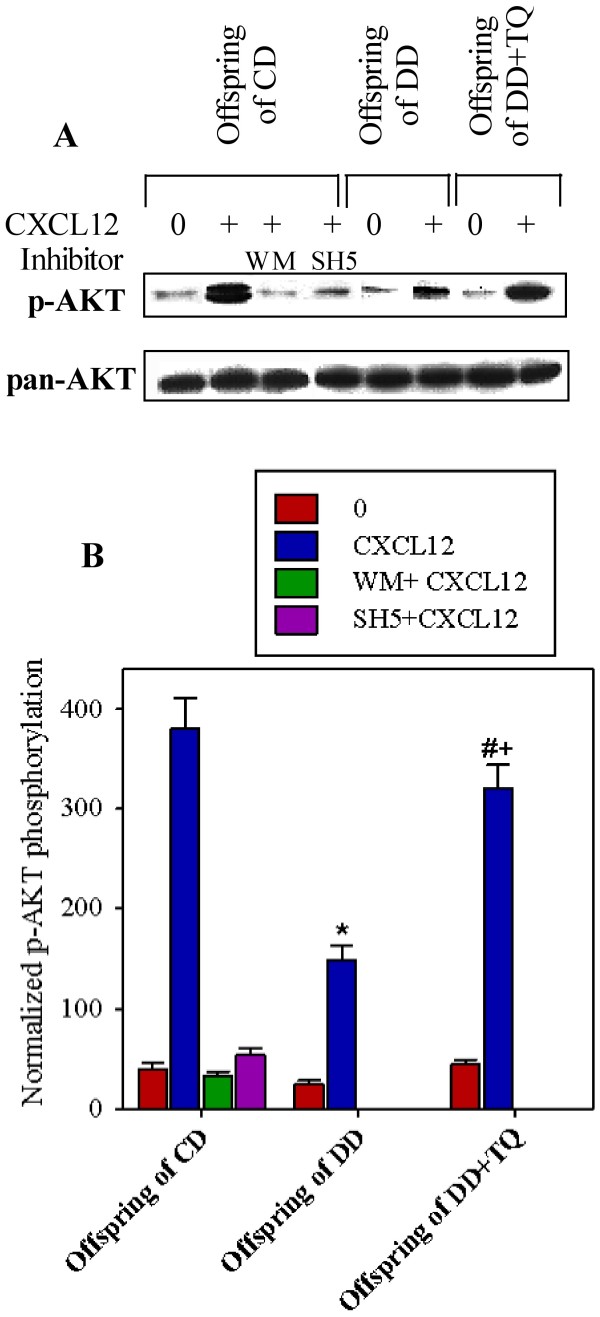
**PI3K/AKT signaling is impaired in the offspring of DD. **The level of phosphorylated AKT (p-AKT), which is part of the main signaling pathway downstream of PI3K, was determined by western blot analysis of PBMCs after stimulation with CXCL12. PBMCs were isolated from the offspring of CD, DD, and DD + TQ and pretreated with medium, WM, and SH5 for 1 h before stimulation with medium (0) or CXCL12 (250 ng/ml) for 5 min. In one representative experiment (**A**), the phosphorylation of AKT was monitored using an anti-p-AKT antibody and normalized against the total relevant protein (pan-AKT) on stripped blots. The experiment was conducted for 5 offspring from each group, and the results are expressed as the mean ± SEM normalized phosphorylation values (**B**). ^*^P < 0.05 for diabetic vs. control; ^#^P < 0.05 for diabetic + TQ vs. diabetic; ^+^P < 0.05 for diabetic + TQ vs. control.

We investigated whether impaired lymphocyte proliferation in the offspring of DD is associated with aberrant PI3K/AKT signaling. PBMCs were isolated from the offspring of CD, DD, and DD treated with TQ, followed by pretreatment with medium, WM, and SH5 for 1 h before stimulation with or without CXCL12 (250 ng/ml) for 5 min. In one representative experiment, CXCL12 induced an obvious phosphorylation of AKT in the PBMCs from the offspring of CD. The PBMCs of the offspring of DD exhibited aberrant phosphorylation of AKT following stimulation with CXCL12. However, the CXCL12-mediated phosphorylation of AKT was clearly restored in the PBMCs of the offspring of DD that were treated with TQ during gestation and lactation. The treatment of PBMCs with WM and SH5 prior to stimulation with CXCL12 markedly inhibited AKT phosphorylation, as shown for the PBMCs from the offspring of DD (Figure 4A). This experiment was conducted with 5 offspring from each group, and the results are expressed as the mean ± SEM of the normalized phosphorylation values (Figure 4B). The level of phosphorylated AKT was normalized to the amount of total AKT. We observed that the CXCL12-mediated phosphorylation of AKT was significantly diminished from 380 ± 31 in the PBMCs from the offspring of CD to 150 ± 13 in the PBMCs from the offspring of DD. Moreover, CXCL12-induced AKT phosphorylation was restored (320 ± 24) in the PBMCs from the offspring of DD treated with TQ.

## Discussion

Diabetes mellitus is associated with many metabolic complications [25]. As a result of these complications, in this study, the pregnancy success rate among the dams was clearly decreased, and significantly fewer neonates were born to diabetic mice compared with control rats. In addition, GD resulted in macrosomic pups with several postpartum complications. For example, at 8 weeks of age, blood glucose and free radical levels (ROS and hydroperoxide) were significantly higher in the plasma of pups born to DD. The complications related to diabetes may result from the generation of free radicals, which damage cellular components such as lipids, proteins, and DNA [26,27]. ROS were elevated in STZ-induced diabetic mice in the present study, as previously demonstrated by Kojo et al. [28]. The number of circulating lymphocytes and insulin levels significantly decreased in pups born to DD compared to offspring born to DD treated with TQ and those born to CD. Furthermore, offspring born to DD exhibited increased lipid levels and were abnormally obese. Interestingly, supplementation of DD with TQ during pregnancy and lactation significantly restored the plasma lipid profiles of their offspring. Increases in blood glucose and plasma fat levels are risk factors for metabolic syndrome and its future complications, such as vascular disease [29]. The modifications induced by maternal diabetes may be attributed to hyperglycemia and fetal hyperinsulinemia, which affect lipid and protein synthesis [30]. Furthermore, maternal hyperglycemia stimulates fetal growth due to the increased availability of glucose in the blood and inappropriate regulation of growth factors, resulting in macrosomic pups [31]. TQ is a promising bioactive phytochemical compound which is found in the seeds of *Nigella sativa* plant. It has recently attracted significant scientific attention due to its potent *in vitro* and *in vivo* anticancer, anti-inflammatory and effective antioxidant properties for normal cells. TQ is well known for scientists where there are more than 400 published works concluding its properties and effects on many different cells including normal and cancer cells. TQ is easily absorbed from the intestine to the blood and then to all the body organs triggering different effects on all the body organs [32]. Because it possesses potent antioxidant properties, supplementation with TQ improves the risk of developing diabetic complications, such as decreasing elevated levels of ROS and MDA (marker of oxidative lipid damage) and regulating plasma concentrations of cholesterol, triglycerides, and glucose [19]. Once it is absorbed from the intestine to the blood, TQ triggers multiple signaling pathways on different organs. We recently demonstrated that TQ ameliorates the immunological and histological changes induced by exposure to imidacloprid insecticide [33]. Our present data demonstrated that TQ ameliorated the aberrant hydroperoxide and ROS levels in pups born to DD. The results indicated a subsequent decrease in the percentage of abortions, an increase in the number of successful pregnancies, and decreased pup mortality. These phenomena may also be mediated by increases in the levels of GST, GSH, and catalase and a decrease in the DNA damage caused by TQ [34]. The results of this work indicate that the use of TQ can be beneficial in preventing and treating hyperglycemia and dyslipidemias in the offspring of diabetic dams. The hypoglycemic effect of TQ in diabetic rats is mediated through a decrease in hepatic gluconeogenesis and glucose production [35]. Thus, TQ affects insulin levels, and this effect has been attributed to the antioxidant activity of TQ, which may alleviate the damage to beta cells in the pancreas caused by STZ [32]. The findings of the current study are consistent with the findings of our previous study that the nutritional supplementation of GD mothers with the natural antioxidant TQ during pregnancy and lactation improves the risk of developing diabetic complications [17]. In the present study, the levels of the pro-inflammatory cytokines IL-1β, IL-6, and TNF-α were significantly elevated in the offspring of DD and were restored by TQ treatment. Similar observations were made in our previous study [17], in which supplementation with TQ was found to have broad anti-inflammatory effects in a diabetic rat model. These results are in agreement with previous studies [36,37], in which TQ was shown to attenuate the levels of pro-inflammatory cytokines. In offspring born to DD, the plasma levels of IL-2 and IL-4 are significantly reduced, providing important evidence of impaired immune function [38]. By contrast, a previous study has revealed that serum IL-4 levels did not vary among gestational diabetic mothers and control mothers or macrosomic babies and control babies [13]. In pups born to DD, we observed a marked decrease in the plasma levels of IL-2 and IL-4, accompanied by a marked reduction in the proliferative capacity of antigen-stimulated lymphocytes. Several studies have revealed that decreased plasma levels of IL-2, which promotes T cell survival and proliferation, occur consistently in several diseases and indicate defective T cell function. In this study, we found that TQ significantly restored both the levels of IL-2 and IL-4 and lymphocyte proliferation. This restorative effect of TQ improved and maintained an efficient immune response of lymphocytes in the offspring, in agreement with our previous work [17]. We previously demonstrated that vitamin C, as an antioxidant agent, reconstitutes polyfunctional T cells and ameliorates diabetic complications in streptozotocin-induced diabetic rats [24]. Furthermore, other investigators demonstrated that combined treatment with vitamin E and vitamin C decreases oxidative stress and improves fetal outcome in experimental diabetic pregnancy [39]. Thus, to compare the effects of TQ with another antioxidant further studies of combinations of TQ and vitamin C during GD are underway. Increased AKT pathway signaling has been shown to be directly correlated with increased rates of glucose metabolism [40]. The PI3K/AKT signaling pathway is important for insulin signaling and glucose metabolism, lymphocyte migration, proliferation, and differentiation, and TGF-β signaling regulation, making it an important therapeutic target for the treatment of cancer, diabetes, and other diseases [41]. Our data demonstrates that the supplementation of DD with TQ during GD restores the impaired PI3K/AKT signaling pathway in the lymphocytes of their offspring. Regulation of the PI3K/AKT signaling pathway by TQ may improve the risk of developing diabetic complications via insulin signaling and glucose metabolism. Although TQ has a wide range of therapeutic benefits, there is lack of clinical evaluation of TQ in humans and the need for clinical studies to assess its health benefits on human is required. Therefore, toxicity studies are essential and a step to the right direction to provide a solid starting point for further preclinical and clinical evaluation of such an important phytochemical compound. We previously explored the effects of TQ on human cancer cells, as well as in mice and rat models [17,42,43]. Before we started to used the TQ in our laboratory, we determined the maximum tolerated dose of TQ for intraperitoneal injection and oral gavages. We found that the maximum tolerated dose was 20 mg/kg in male and 15 mg/kg in female rodent, whereas for oral administration it was 150 mg/kg in both male and female rats. Therefore, we used a low dose of TQ that has no toxicity effects even it administered for up to 3 months. In the present study, a low dose of TQ (20 mg/kg body weight/day) was administered during gestation and lactation periods. Taken together, our data suggest that the use of TQ is a potential strategy for the prevention of DM, abnormal lipid profiles and their complications, and to treat these diseases by preventing the development of diabetic complications and maintaining an efficient lymphocyte immune response in offspring later in life.

## Abbreviations

(CD): Control dams; (DD): Diabetic dams; (DM): Diabetes mellitus; (HDL-C): High density lipoprotein-cholesterol; (IL): Interleukin; (LDL-C): Low density lipoprotein-cholesterol; (MDM): Maternal diabetes mellitus; (ROS): Reactive oxygen species; (STZ): Streptozotocin; (TQ): Thymoquinone; (TNF-α): Tumor necrosis factor-alpha.

## Competing interests

The authors declare no conflicts of interest. This manuscript has not been published or submitted elsewhere. The work complies with the Ethical Policies of the Journal and has been conducted under internationally accepted ethical standards after relevant ethical review.

## Authors’ contributions

GB put the design of the study, carried out the immunological assays, prepared figures, drafted the manuscript and performed the statistical analysis. MHM was responsible for the nutrition of the animal model and participated in drafting the manuscript. KF was responsible for the data and statistical analysis. HW participated in the data analysis. OZ participated in the sample collections. DMR participated in the data analysis and drafting the manuscript. All authors read and approved the final manuscript.
